# Coastal Halophytes: Potent Source of Bioactive Molecules from Saline Environment

**DOI:** 10.3390/plants12091857

**Published:** 2023-04-30

**Authors:** Milan Stanković, Zorica Stojanović-Radić, Dragana Jakovljević, Nenad Zlatić, Milica Luković, Zora Dajić-Stevanović

**Affiliations:** 1 Department of Biology and Ecology, Faculty of Science, University of Kragujevac, 34000 Kragujevac, Serbia; mstankovic@kg.ac.rs (M.S.);; 2 Department of Biology and Ecology, Faculty of Sciences and Mathematics, University of Niš, 18000 Niš, Serbia; 3 Department of Natural Sciences, Faculty of Hotel Management and Tourism in Vrnjačka Banja, University of Kragujevac, 36210 Vrnjačka Banja, Serbia; 4 Department of Botany, Faculty of Agriculture, University of Belgrade, 11080 Belgrade, Serbia

**Keywords:** Southeast Mediterranean halophytes, saline habitats, secondary metabolites, antioxidant activity, antimicrobial activity

## Abstract

This study represents a comparative analysis of secondary metabolites content, antioxidant, and antimicrobial activity of 24 halophytes from coastal saline habitats of the Balkan Peninsula (Montenegro, Albania, and Greece). Total content of phenolics, flavonoids, tannins, anthocyanins, antioxidant, and antimicrobial activity was determined for dry methanolic (DME) and crude water extracts (CWE) and compared with well-known medicinal plants. The total phenolic content ranged from 13.23 to 376.08 mg of GA/g of DME, and from 33.68 to 511.10 mg/mL of CWE. The content of flavonoids ranged from 12.63 to 77.36 mg of RU/g of DME, and from 12.13 to 26.35 mg/mL of CWE. Total tannins and anthocyanins varied from 0.05 to 2.44 mg/mL, and from 1.31 to 39.81 µg/L, respectively. The antioxidant activity ranged from 1147.68 to 15.02 µg/mL for DME and from 1613.05 to 21.96 µg/mL for CWE. The best antioxidant properties, and the highest content of phenolic compounds, were determined for *Polygonum maritimum* and *Limonium vulgare* with values similar to or higher compared to the medicinal plants. Halophytes with significant antimicrobial potential were *Limonium vulgare*, *L. angustifolium*, and *Artemisia maritima*. Some of the analyzed coastal halophytes can be considered rich natural sources of phenolic compounds, with favorable antioxidative and antimicrobial properties.

## 1. Introduction

Salinity is one of the leading problems in coastal areas due to the impact of natural and anthropogenic factors that strongly influence the distribution of plants in certain regions. Approximately 400 million hectares of land are currently affected by salinity, whereas 20% of the arable land in the world and half of the irrigated soils are exposed to increased salt concentrations in the substrate. Most plant species do not have the ability to tolerate high concentrations of salt in the substrate, and consequently they cannot grow on saline habitats. However, plants known as salt tolerant—the halophytes— have developed various mechanisms to cope with salinity stress [1,2].

Halophytes are plant species native to habitats with increased concentration of salt in soil. They can either be separated into inland, coastal, or near coastal species depending on the type of habitat they occupy, i.e., upon the proximity to the open sea [2,3]. There are more than 2500 halophyte species with different mechanisms of adaptation to saline habitats. Some plant families such as Amaranthaceae, Plumbaginaceae, Poaceae, and Asteraceae contain a significant number of salt tolerant species, used as foods, medicinal plants, ornamentals, fodder, fuels, source of fiber, highly nutritious oilseeds, biomass, etc. [4,5,6]. In rural areas, medicinal plants have a significant biological activity that has been shown to treat a wide range of illnesses and bacterial infections. Additionally, in both in vitro and in vivo conditions, their active metabolites exhibit antimicrobial, antiviral, antiproliferative, and antioxidant action [1,7].

The powerful biological capacities of halophytes are related to their ability to produce secondary metabolites (mainly phenolic compounds) to overcome harsh environmental conditions and salinity-induced production of reactive oxygen species such as hydrogen peroxide, hydroxyl radicals, and superoxide anions [8]. Therefore, in recent decades, halophytes are recognized as valid sources of polyphenols and other secondary metabolites with strong antioxidant activity. Natural bioactive molecules derived from halophytes are significant in various areas of application because they are highly effective in preventing oxidative stress.

In our previous study [9], we pointed out the lack of studies on European halophytes as medicinal plants, and their potential as a source of bioactive compounds whose increased biosynthesis is an adaptive response to salt stress. In this study, twenty-four halophytic species collected from different coastal saline habitats of the Balkan Peninsula (territory of Montenegro, Albania, and Greece) were studied for their antioxidant and antimicrobial activities in relation to the content of a major group of phenolic compounds (total phenolics, flavonoids, tannins, and anthocyanins).

## 2. Results and Discussion

### 2.1. Total Phenolic Content

The quantitative and qualitative composition and activity of phenolic compounds depend on various factors including taxonomy, environmental and developmental factors, as well as methods of extraction [9]. The tolerance of halophytes to salinity stress comes, among others, from the modulation of physiological and biochemical attributes related to ion toxicity and oxidative stress, whereas the metabolite level is directly related to saline conditions and substrate salinity [10,11,12].

The results of the total amount of phenolic compounds in dry methanolic extracts (DME) and crude water extracts (CWE) of above-ground plant parts of 24 tested species are shown in Table 1. Total phenolic content in DME varies from 13.23 to 376.08 mg of GA/g. The highest concentrations of total phenols (significantly higher compared to all tested plants) have been observed in extracts of *Polygonum maritimum* (376.08 mg of GA/g). High concentrations of total phenols were also measured in extracts from *L. vulgare* (266.80) followed by *E. peplus* (186.73), *A. tinctoria* (164.42), *L. angustifolium* (135.68), and *V. agnus-castus* (109.46). Compared to all tested species, a significantly lower concentration of total phenolic compounds was determined for DME of *M. marina*, *S. fruticosa*, and *H. portulacoides*.

The total phenolic content in the CWE of tested halophytes varied from 33.68 to 511.10 mg of GA/mL (Table 1). The highest concentration of total phenols, significantly higher than all other tested plants, was observed in *L. vulgare* (511.10 mg/mL). High concentrations of total phenols were also measured in extracts from *P. maritimum* (363.08), *E. peplus* (338.60), *A. maritima* (277.78), *A. tinctoria* (262.88), *L angustifolium* (233.89), *V. agnus-castus* (216.96), and *E. spinosa* (210.27). The lowest concentration of total phenol compounds was determined for the CWE of the species *S. kali*—Gr and *M. marina* (37.57 and 36.68, respectively), significantly lower compared to other tested species.

Based on the results shown for the total amount of phenolic compounds in DME and CWE, as well as the results obtained from the comparative study of selected plant species (*C. sinensis, S. officinalis, L. officinalis*, and *O. europaea*) presented in Table 1, it has been shown that individual halophytes possess a higher or similar number of phenols compared to the tested commercial species.

High content of phenolic compounds in DME and CWE of halophytes is related to the available biologically active substances, which, depending on the polarity of the solvents, differently dissolve in the methanol and water, respectively. The polarity of solvents used for extraction affects the extraction efficiency of phenolic compounds whereby less polar solvents extract a smaller number of phenolic compounds [13]. The least polar solvents are generally considered as suitable for the extraction of lipophilic phenols, and polar solvents are better for hydrophilic phenols [14]. In accord, *Polygonum maritimum* has, compared to *C. sinensis*, a significantly higher content of phenolic compounds in the DME, and lower in the CWE. The latter is not true for *L. vulgare* since this halophytic species exhibits a reversed pattern.

The results showed a wide variation of phenolic content among the studied species, as well as different variation among species of the same family, since significant variability between the species of Amaranthaceae family can be seen (*S. kali* from Montenegro, Albania, or Greece, *S. soda*, *S. europaea*, *S*. *fruticosa*, *H. portulacoides*, and *H. strobilaceum*). Another example of the mentioned variability is in Plumbaginaceae (*L. angustifolium* and *L. vulgare*), Apiaceae (*E. spinosa* and *E. maritimum*), and Asteraceae (*A. maritima* and *X. italicum*) family. The results show that plant phylogeny does not reflect the amount of total phenolics [15]. The differences in the concentration of phenols in the studied species are associated with various molecular, physiological, phylogenetic, and morphological structures that emerge as a response to the influence of environmental factors [16], increasing under increased salt concentrations and drought [17]. High content of total phenolic compounds of the *Limonium* and *Polygonum* species, including flavonoids, is well documented [18,19,20,21,22].

### 2.2. Flavonoid Concentration

The main characteristic of flavonoids is the presence of two benzene rings associated with an aliphatic sequence. The changes in the oxidation state of aliphatic sequence, together with the diversity of substituents, leads to a wide variety of structurally different flavonoids followed by changes in solubility, stability, and ability to neutralize free radicals [8,23].

The results of the total flavonoid concentrations in DME and CWE of investigated species are shown in Table 1. The concentration of total flavonoids in examined DME of halophytes varies from 12.63 to 77.36 mg of Ru/g. The highest concentration of flavonoids has been observed in *V. agnus-castus* (77.36 mg of Ru/g), which is significantly higher compared to other tested plants. High concentrations of flavonoids were also measured in extracts from *S. kali*-Al (69.47) and *S. kali*-Mn (66.53), as well as in *L. angustifolium* (61.25), *L. vulgare* (60.69), and *E. peplus* (58.34). Compared to all tested species, a significantly lower concentration of flavonoid compounds was determined for *S. fruticosa* and *H. portulacoides*.

The concentration of the total flavonoids in the CWE of tested halophytes varied from 12.13 to 26.35 mg/mL. The highest concentrations were observed in CWE of *L. vulgare* (26.35 mg/mL) and *V. agnus-castus* (26.10). The obtained values were significantly different compared to other tested species including medicinal plants used for comparison. High concentrations of flavonoids were also measured in *C. soldanella* (23.80), *E. peplus* (23.58), *S. soda*—Mn (22.29), *C. maritima* (22.29), *A. maritima* (21.16), *P. maritimum* (20.53), and *L. angustifolium* (20.37). A significantly lower concentration of flavonoids was determined for CWE of *T. terrestris* and *S. europaea*.

Based on the results for the concentration of flavonoids in DME and CWE, as well as the results obtained from the comparative study of selected plant species (*C. sinensis, S. officinalis, L. officinalis*, and *O. europaea*) presented in Table 1, it has been shown that the species *V. agnus-castus*, *S. kali*-Al, and *S. kali*-Mn possess significantly higher concentrations of flavonoids in their DME compared to *C. sinensis*, which contains the highest concentrations of flavonoids among the species chosen for comparison. *L. vulgare*, *V. agnus-castus*, *C. soldanella*, *E. peplus*, *S. soda*, and *C. maritima* had significantly higher concentrations of flavonoids in CWE than the flavonoid-richest commercial plant, the sage. According to the presented results, DME possess higher concentrations of flavonoids compared to CWE, suggesting that these compounds are better dissolved in less polar solvents.

The results also show that there is a certain degree of variation in the concentration of flavonoids among the investigated species and the families to which they belong. Significant variability has been observed for DME and CWE of species belonging to the families Amaranthaceae (*S. kali* from Montenegro, Albania, or Greece, *S. soda*, *S. europaea*, *S*. *fruticosa*, *H. portulacoides*, and *H. strobilaceum*) and in CWE of species belonging to the families Apiaceae (*E. spinosa* and *E. maritimum*) and Asteraceae (*A. maritima* and *X. italicum*). There was no significant difference in the concentration of flavonoids in DWE of species belonging to the families Plumbaginaceae and Apiaceae. Observed differences in the total flavonoid concentration in investigated coastal halophytes may relate to different phylogenetic pathways of specific taxa. Previous studies have confirmed that certain plants from salinity habitats synthesize higher levels of flavonoids to adapt to an increased salt level in the substrate [9]. In accordance with our findings, Kımna and Fafal [24] demonstrated that *V. agnus-castus* extracts are rich in flavonoids; still, extracts prepared with different methods contain different amounts of flavonoid components. Significant concentrations of total flavonoids have been determined by several genera and species of coastal halophytes such as *Salsola* [25], *Limoniastrum* [26,27], *Tribulus terestris* [28] and *Suaeda fruticosa* [29].

### 2.3. Tannin Content

Tannins are natural polyphenolic compounds commonly found in plants and their parts (e.g., bark, fruit, and seeds). The number of tannins in plants is important because, like many polyphenolic compounds, they exhibit high biological activity, and as a natural antioxidant, their amount in the plant extract may contribute to its better antioxidative activity [30]. Tannins are highly soluble in polar solvents [31], indicating that the best yield of tannins in the extract should be achieved using polar organic solvents. The results of the total amount of tannins of investigated species are shown in Table 2. The concentration of total tannins in extracts from examined halophytes varied from 0.05 to 3.45 mg/mL of DME. The highest concentrations of total tannins were observed in extracts of species *X. italicum* (3.50 mg/mL), followed by *V. agnus-castus* (2.47) and *T. terrestris* (2.36). High concentrations of total tannins were measured in *E. peplus* (1.77), *C. maritimum* (1.40), and *E. maritimum* (1.15). The lowest concentration of total tannins was determined for extracts of species *S. fruticosa* and *H. portulacoides*. Based on the obtained results for the total amount of tannins in the extracts of coastal halophytes, as well as the results obtained from the comparative study of selected commercial plants (Table 2), it can be added that the species *X. italicum* possesses significantly higher content of total tannins in the extract compared to that of the *C. sinensis*, which showed the highest tannin content among surveyed commercial herbs. According to Haque et al. [32], *X. italicum* is used in traditional medicine and extracts of this species possess various biological activities. The presence of tannins in various extracts of *X. italicum* is confirmed together with cytotoxic and antibacterial activities of this plant species [33].

### 2.4. Anthocyanin Content

Anthocyanins are herbal pigments that are synthesized via phenylpropanoid pathway and belong to the flavonoid group. Their occurrence in plants exposed to saline habitats has already been described as an adaptive response to stress conditions [34]. The results of the total concentration of anthocyanins in extracts of investigated species are shown in Table 2. The concentration of total anthocyanins in examined species varied from 1.41 to 39.32 μg/mL of DME. The highest concentration of total anthocyanins was observed in *E. peplus* (39.32 μg/mL). The species with high concentrations of total anthocyanins were *C. soldanella* (31.22) and *A. tinctoria* (30.90). The lowest concentration of total anthocyanins was determined for extracts of the species *Polygonum maritimum* and *C. maritima*. Based on the results shown for the total concentration of anthocyanins in extracts in coastal halophytes, and the results obtained from the comparative study of selected commercial plants (Table 2), it can be noticed that certain types of halophytes have a significantly higher concentration of anthocyanins. For example, the species *E. peplus* has two and a half times higher concentration of anthocyanins than species *C. sinensis* and four times higher concentration of identical compounds than species *L. officinalis,* the latter two being the highest ranked among the plants used for comparison. Also, *C. soldanella* and *A. tinctoria* possess two times higher concentrations of anthocyanins than green tea extracts, which have the highest concentrations of anthocyanins in the extracts among the investigated commercial plants. Plants from the *Euphorbia* genus are well known for their therapeutic activities and applications in traditional medicine, and new insights testify about high content of polyphenolic compounds [35]. There is an increasing interest in extracts from *E. peplus* due to various biological activities and for the synthesis of nanoparticles [36,37,38]. Additionally, it has been reported that various compounds, including anthocyanins, isolated from *C. soldanella* extracts, exert different physiological activities [39].

### 2.5. Antioxidant Activity

The results of antioxidant activity in DME and CWE of above-ground plant parts of investigated species are shown in Table 3, whereby lower values identify species that have higher antioxidant activity. The antioxidant activity of the DME of the investigated halophytes varied from 1147.68 to 15.02 μg/mL. The highest ability of neutralizing free radicals, significantly higher from other tested halophytes, was observed for *Polygonum maritimum* (15.02) and *L. vulgare* (15.52). High antioxidant potential is also exhibited by *E. peplus* (29.19), *A. tinctoria* (52.86), and *L. angustifolium* (58.66). Poor neutralization value for free radicals and the lowest antioxidant potential was demonstrated for *M. marina* and *H. portulacoides*.

The antioxidative activity of CWE of tested species varied from 1613.05 to 21.96 μg/mL. The highest antioxidant potential is present in *L. vulgare* (21.96), *Polygonum mari-timum* (24.59) and *E. peplus* (26.98). The great ability of neutralizing free radicals was ob-served in *L. angustifolium* (42.12). Species that showed good to moderate activity were *A. maritima* (80.23), *A. tinctoria* (81.27), *E. spinosa* (86.47), *C. soldanella* (145.11), *V. agnus-castus* (165.79) and *E. maritimum* (192.33). The rest of the examined plants show a weak neutral-ization value for free radicals, while the lowest antioxidant potential has been identified in *M. marina* and *H. portulacoides*. 

Based on the results shown for the antioxidant activity in DME and CWE, as well the results obtained from the comparative study of selected commercial plants (Table 3), it can be observed that the antioxidant activity of particular halophytic species both in DME and CWE are very similar or higher comparing to the most-used commercially available plants. It should be noticed that both DME and CWE of species *L. vulgare, Polygonum mari-timum*, and *E. peplus* show similar neutralization capacity of free radicals as DME and CWE of green tea, which is best ranked among species for comparison.

High antioxidant activity found in some halophyte plants could be related to their biologically active compounds. A comparative analysis conducted in recent years regarding the content of phenolic compounds and antioxidant activity of extracts of plants from saline habitats confirms the connection between these two parameters, since, in general, better antioxidant activity is related to high content of phenolic compounds. However, since the specific conditions of particular saline habitat cause the specific metabolic response of plant species, various factors must be considered [40]. In the most recent study, Carius et al. [41] analyzed the chemical profile of aerial parts of *L. vulgare* collected in Portugal and demonstrated a richness in polyphenolic compounds, but with differences among plant parts and stage of development. Additionally, investigation of antioxidant activities demonstrated variability among the *Limonium* species in relation to the environmental conditions of their natural biotopes, whereas when comparing eight *Limonium* species from Tunisia including *L. boitardii*, *L. delicatulum*, *L. densiflorum*, *L. ferulaceum*, *L. spathulatum*, *L. tunetanum*, *L. virgatum*, and *L. vulgare*, species *L. vulgare* was the one with the highest antioxidant capacity [42].

### 2.6. Antimicrobial Activity

Antimicrobial activity of twenty-four coastal halophytes was tested against three Gram-positive, three Gram-negative, and one fungal strain and the results are shown in Table 4. According to collected data, values of MIC of the tested extracts generally varied within the range from 0.15 mg/mL to 20 mg/mL. The strongest antimicrobial activity was observed for the extracts of species *L. vulgare* and *L. angustifolium,* followed by *A. maritima*, *E. peplus*, *A. tinctoria*, and *V. agnus-castus*. Results on antimicrobial activity of the studied plants pointed to notably higher activity of all extracts against Gram-positive bacteria. Among the tested Gram-positive bacteria, *B. cereus* showed the highest susceptibility with MIC values ranging from 0.15 to 10.00 mg/mL. On the other hand, the lowest antimicrobial effects were shown on *K. oxytoca*, followed by species *S. lutea* among the tested Gram-negative bacteria. Fungal strain *A. brasiliensis* shows moderate resistance to extracts.

Based on the presented results of the halophytes extracts for antimicrobial activity, as well as the results from tested standards (Table 4), it can be noticed that the species *L. vulgare* and *L. angustifolium* possess a higher antimicrobial activity compared to the *S. officinalis*, the best ranked among commercial plants. In addition, *A. maritima, E. peplus, A. tinctoria*, and *V. agnus-castus* have better antimicrobial activity than all other commercial plants studied for comparison. When it comes to literature data regarding the antimicro-bial activity of halophytes the information is scarce. Lopes et al. [43] reviewed potential application of different halophyte species as antimicrobial agents and pointed out that the great impact on the final effects of extracts has the type of phenolic compounds present in the extract and/or the synergistic action among different phenolic compounds and other types of molecules with antimicrobial action from the extract. According to these authors, plant extracts with MIC values of less than 250 µg/mL are of great relevance. Based on this information and according to the results of this study, species *L. vulgare*, *L. angustifolium*, *E. peplus*, *V. agnus-castus* and *S. kali* can be regarded as natural antibacterial agents. Among mentioned species, a special attention of future studies should be given to *L. vulgare* and *L. angustifolium*, since these two species showed non-selective activity and were highly efficient against both Gram-positive, Gram-negative, and fungal strains.

### 2.7. Correlation, Principal Component and Cluster Analysis

#### 2.7.1. Pearson’s Correlation

**Figure 1:**
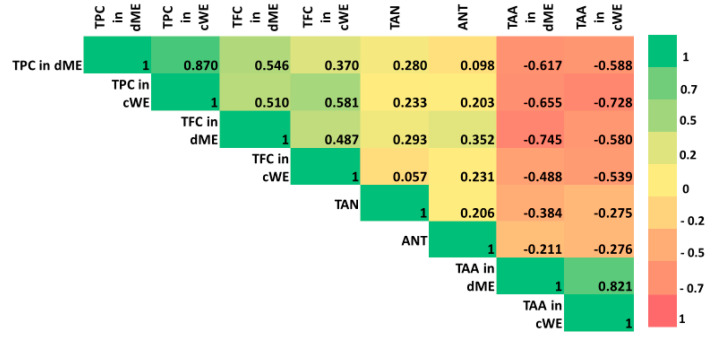
Pearson’s correlation among tested traits presented as heat map. TPC—total phenolic content, dME—dry methanol extracts, cWE—crude water extract, TFC—total flavonoid concentration, TAA—total antioxidant activity, TAN—tannins, ANT—anthocyanins.

Pearson’s correlation analysis was conducted to view the variations between various coastal halophytic species and their association to metabolite contents and antioxidant activity. The Pearson’s correlation analysis presented using heat map (Figure 1) showed important associations were for both DME and CWE strong negative correlation was found between DPPH-radical scavenging activity (total antioxidant activity—TAA), total phenolic content, and flavonoid concentration. Considering that TAA is expressed as IC_50_ value (lower values mean higher antioxidant activity), the negative correlation is expected. On the other hand, the weak correlation was found between tannins and total antioxidant activity, as well as between anthocyanins and antioxidant activity for both DME and CWE, emphasizing the major interactions among extract composition and antioxidant properties.

#### 2.7.2. Principal Component Analysis and Hierarchical Clustering

**Figure 2:**
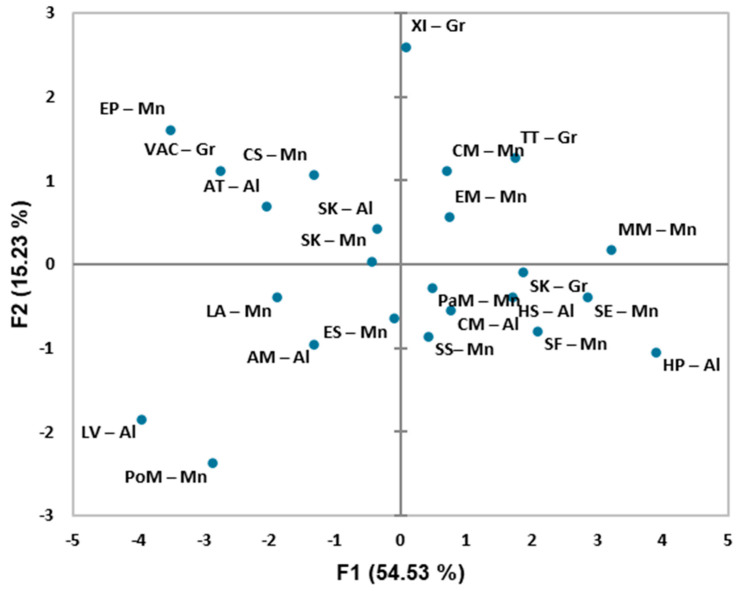
Score plot of halophyte species obtained from principal component analysis. Species are presented as first letters of their Latin names (list is in Table 5).

**Figure 3:**
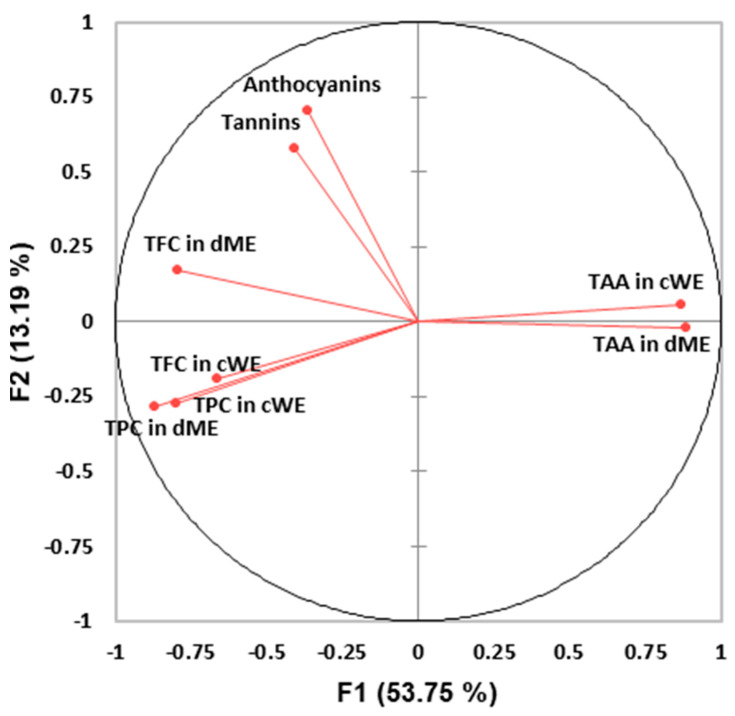
Loading plot of variables obtained from principal component analysis. TPC—total phenolic content, TFC—total flavonoid concentration, TAA—total antioxidant activity, TAN—tannins, ANT—anthocyanins, cWE—crude water extract, dME—dry methanol extracts.

Although the principal component analysis (PCA) distribution could differentiate halophytes based on the tested characteristics, no clear clusters were detected among 24 halophytes (Figure 2). The first two principal components together explained 66.94% of the total information, where the first explained 53.75% and the second explained 13.19% of the total variance. From the PCA, it can be seen that the anthocyanins and tannins are away from all other components indicating that they differ from other tested biochemical characteristics (Figure 3). TPC in DME and TPC in CWE are found in opposition to TAA in DME and TAA in CWE. This is followed by the location of *L. vulgare* (LV—Al) and *Polygonum maritimum* (PoM—Mn) on Figure 2 which can be explained by their high values of phenolics, while the opposite position of *M. marina* (MM—Mn) and *S. europeaea* (SE—Mn) on Figure 2 can be explained by the high IC_50_ values (i.e., low antioxidant activity).

### 2.8. Cluster Analysis of Antioxidant Properties

**Figure 4:**
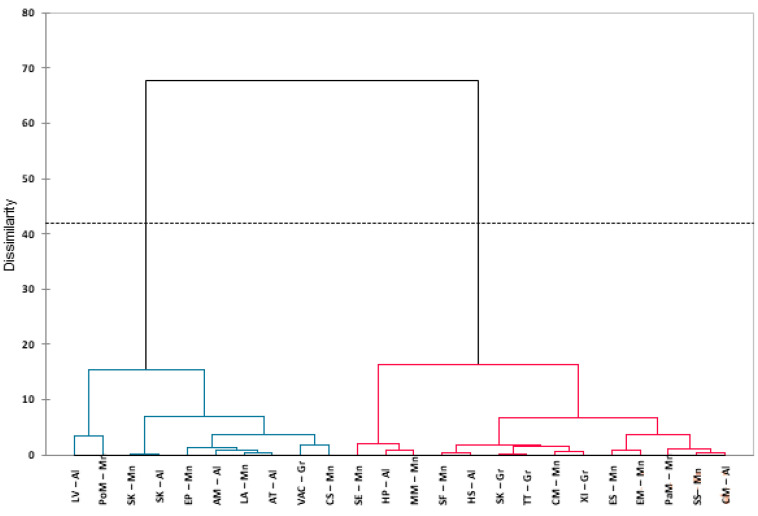
AHC dendrogram based on a Euclidean dissimilarity distance matrix of antioxidant activity, total phenolic content, and flavonoid concentration of 24 halophytic species from different coastal saline habitats of Balkan Peninsula. Blue lines—clade I, red lines—clade II.

Agglomerate Hierarchical Clustering (AHC) was done upon the Euclidean dissimilarity distance matrix which included the total phenolic content, flavonoid concentration, and antioxidant activity data. The dendrogram (Figure 4) shows the presence of two clades that are neither grouped according to their taxonomy nor geographical distribution. Clade I consisted of 14 halophytic species including *Pancratium maritim*—Mn, *S. kali*—Gr, *S. soda*—Mn, *S. europaea*—Mn, *S. fruticosa*—Mn, *H. portulacoides*—Al, *H. strobilaceum*—Al, *T. terrestris*—Gr, *M. marina*—Mn, *C. maritima*—Al, *C. maritimum*—Mn, *E. spinosa*—Mn, *E. maritimum*—Mn, and *X. italicum*—Gr. Clade II is comprised of 10 halophytic species including *S. kali*—Mn, *S. kali*—Al, *L. angustifolium*—Mn, *L. vulgare*—Al, *Polygonum maritimum*—Mn, *E. peplus*—Mn, *A. tinctoria*—Al, *V. agnus-cactus*—Gr, *C. soldanella*—Mn, and *A. maritima*—Al.

Ecological factors of saline habitats include numerous stressful conditions resulting from disturbed mineral and water regimes. A small number of plants are adapted to these conditions because the main problem is overcoming the toxic effects of salt and maintaining water balance under physiological drought conditions. Halophytes are a specific ecological group of plants adapted to salt stress conditions, and the adaptive response involves several structural and physiological strategies. Production of secondary metabolites with different quantitative and qualitative composition is one of the most important mechanisms of stress tolerance [6,34]. Extreme conditions per se affect the antioxidant system of plants, leading to an increase in the production of reactive oxygen forms. Due to the negative effects of abiotic factors, halophytes synthesize bioactive compounds that perform the function of protection in order to neutralize reactive oxygen species. In such habitats, flooding, drought, and substrate salinization have a synergistic effect that multiplies stress in plants and significantly increases the synthesis of compounds with antioxidant activity. This also plays a role in the protection of plant cells and tissues from heat and excessive radiation and stimulation of the endogenous system of antioxidant enzymes. This fact is the reason many halophytes are considered medicinal plants, while others are the subject of research as potential sources of valuable bioactive compounds with medicinal applications [40].

Based on previous studies of main components of secondary metabolites present in the coastal halophytes, diverse groups of bioactive substances were determined, such as phenolics (phenolic acids, flavonoids, coumarins, tannins), terpenoids (mono-, di-, tri- sesquiterpenes), alkaloids, essential oils, etc. [4,44,45,46]. Significant biological activity of different phenolic acids and flavonoids of halophytes has already been confirmed in relation to their antioxidant activity [47]. The results obtained in this study indicate that the high value for the antioxidant and antimicrobial activity of investigated extracts is related to the high content of phenolic compounds. According to Pilluza and Bullita [48], the dependence of the antioxidant activity on the amount of total phenolic compounds is well known and was confirmed by numerous analyses of biological activity of plant extracts.

The presented results of the comparative analysis indicate that the studied halophytes are a source of potent bioactive compounds, but that the quantitative composition and biological activity varies depending on the species and habitat conditions. Such variability was also noted during previous comparative analyses of species from the same family or genera, as well as between samples of the same species sampled from different halophytic habitats. Bearing in mind the ecological value of plant bioactive compounds, as well as the direct relation of environmental conditions and the intensity of secondary metabolism, it is clear that variability is a natural consequence of the complex action of exogenous and endogenous factors [49]. On this basis, expressed biological and ecological differences between the studied species determine differences in quantitative and qualitative characteristics of the active substances. The shown variability in the results of the examined halophytes can be attributed to the complexity of ecological factors that affect coastal habitats. On the other hand, biological activity depends not only on the number of secondary metabolites, but also on their structural characteristics, whereby total phenolic compounds, content of flavonoids, tannins, and anthocyanins, in addition to biological activity analyses, indicates that the representatives of each group vary.

We tested insufficiently studied coastal halophytes from the southeast Balkan, i.e., the Mediterranean part of the Balkan peninsula, comprising the territory of Montenegro, Albania, and Greece, focusing on production of their bioactive compounds and related biological activity. Among the tested 24 halophytic species, *Polygonum maritimum* and *L. vulgare* showed high value of the total phenolic content, as well as the concentration of flavonoids and antioxidant activity. The species of genus *Limonium*—*L. vulgare* and *L. angustifolium* demonstrated significant antimicrobial activities against Gram-positive bacteria, especially towards *B. cereus*. The highest content of tannins and anthocyanins was recorded for *X. italicum*, *V. agnus-cactus*, and *E. peplus*. Moreover, the content of secondary metabolites together with antioxidant and antimicrobial activities of named halophytes was higher compared to the tea plant, common sage, lavender, and olive—commercial plant species well known for their antioxidant and antimicrobial properties. All this leads to the conclusion that investigations of halophytes are reasonable in terms of identification and isolation of potent bioactive compounds. Further studies on the phytochemistry and biological activity of coastal halophytes are needed for understanding the impact of salt stress on biosynthesis pathways and production of specific bioactive metabolites.

## 3. Materials and Methods

### 3.1. Plant Material

Twenty-four halophyte species were sampled at the full flowering stage from their natural habitats situated along the coastal part of the southeast (Montenegro, Albania, and Greece, Table 5). Voucher specimens and herbarium collections are reposited at the Faculty of Agriculture, University of Belgrade (Dept. for Agribotany).

The collected plant specimens were determined at the Faculty of Agriculture, University of Belgrade, following Med-Checklist (a critical inventory of vascular plants of the circum-Mediterranean countries, http://ww2.bgbm.org/mcl/ (accessed on 14 February 2023)).

In addition, four well-known commercial medicinal plants were used for comparison and evaluation of obtained results to assess the bioactive potential of studied native halophytes. The commercial herbs included green tea (*Camelia sinensis* (L.) Kuntze cultivar ‘Yabukita’, voucher specimen 139/22), common sage (*Salvia officinalis* L. cultivar ‘Extrakta’ voucher specimen 140/22), lavender (*Lavandula officinalis* Chaix ex Vill. cultivar ‘Munstead’ voucher specimen 141/22) and olive (*Olea europaea* L. cultivar ‘Amfissa’ voucher specimen 142/22) which were obtained from commercial sources. Voucher specimens and herbarium collections are reposited at the Faculty of Science, University of Kragujevac (Dept. of Biology and Ecology). The above-ground plant parts were dried in the dark at room temperature. Dry samples were ground in a blender and kept in dark vials until analysis.

### 3.2. Preparation of Plant Extracts

For dry methanol extract (DME) preparation, the plant material (10 g) was transferred into dark colored flasks filled with 200 mL of pure methanol and stored at room temperature. After 48 h, material was filtered using Whatman No. 1 filter paper and residues were re-extracted with an equal volume of solvents. Combined supernatants were evaporated to dryness under vacuum at 40 °C using rotary evaporator (IKA RV 10 digital V-C, IKA^®^-Werke Gmbh & Co. KG, Mindelheim, Germany). The obtained extracts were kept in sterile sample tubes and stored in a refrigerator at 4 °C. To determine the total content of analyzed phenolic compounds in water extracts (CWE), 100 mL of boiling distilled water was mixed with 1 g of the prepared dry plant material. The mixture was left to cool at room temperature for 10 min and then filtered to obtain a clear solution for further processing [50].

### 3.3. Chemicals

Organic solvent and sodium hydrogen carbonate were purchased from Zorka pharma Šabac, Serbia. Sodium metabisulfite were purchased from MosLab Belgrade, Serbia. 2,2-dyphenyl-1-picrylhydrazyl (DPPH) was obtained from Sigma Chemicals Co., St. Louis, MO, USA. Folin–Ciocalteu phenol reagent, 3-tert-butyl-4-hydroxyanisole (BHA), and aluminium chloride hexahydrate (AlCl_3_ × 6H_2_O) were purchased from Fluka Chemie AG, Buchs, Switzerland. All other solvents and chemicals were of analytical grade.

### 3.4. Determination of Total Phenolic Content

The total phenolic content was determined using the spectrophotometric method with 2.5 mL of 10% Folin–Ciocalteu reagent dissolved in water, and 2.5 mL 7.5% NaHCO_3_ were mixed with 0.5 mL of DME (1 mg/mL) or CWE to obtain the reaction mixture [51]. The samples were further incubated at 45 °C for 15 min. The absorbance was measured at λ_max_ = 765 nm (spectrophotometer Jenway UV/VIS 6105 with the resolution range 1 nm). The samples were prepared in triplicate and the mean value of absorbance was obtained. The blank was prepared with pure methanol solution. The same procedure was repeated for the gallic acid, and the calibration curve was constructed. The total phenolic content was expressed as gallic acid equivalent (mg of GA/g of DME or mg/mL of CWE, respectively).

### 3.5. Determination of Flavonoid Content

The DME samples contained 1 mL of methanol solution of the extract in the concentration of 1 mg/mL and 1 mL of 2% AlCl_3_ solution dissolved in methanol. The second group of samples contained 1 mL of CWE and 1 mL of 2% AlCl_3_ solution dissolved in the methanol [52]. The absorbance was measured with the spectrophotometer Jenway UV/VIS 6105 with the resolution range 1 nm. The samples were prepared in triplicate and the mean value of absorbance was obtained. The same procedure was repeated for the rutin, and the calibration line was constructed. Concentration of flavonoids was expressed in terms of rutin equivalent (mg of Ru/g of DME or mg of Ru/mL of CWE, respectively).

### 3.6. Determination of Total Tannins Content

The total tannins content was determined using the spectrophotometric method [53] with slight modifications. In order to determine the total content of tannins, two samples containing 2 mL of ME extract, 3 mL of concentrated HCl, and 1 mL of distilled water were used. The first sample was incubated at 100 °C for 30 min, whereas 0.5 mL ethanol was added into the second sample. Absorbances of both samples were measured at λ_max_ = 470, 520, and 570 nm. The differences (ΔA) between the samples measured at the same wavelength (ΔA470, ΔA520, ΔA570) were calculated. Hence, taking into account the ΔA470, ΔA520, ΔA570, the values were calculated using the following equations: ΔA520 = 1.1 × ΔA470; ΔA520 = 1.54 × ΔA570. Total tannins content expressed as g/L of extract was calculated as follows: TTC (g/L) = 15.7 × minimum (ΔA520).

### 3.7. Determination of Anthocyanins Content

Two samples containing 0.5 mL of DME, 0.5 mL solution of 0.1% HCl in ethanol and 10 mL of 2% HCl aqueous solution were used to obtain the value of total content of anthocyanins. Distilled water (4.4 mL) was added into one sample, whereas 4.4 mL of 13% sodium bisulfite solution was added into the other sample. The 1:1 dilution of the mixture was made. Sample absorbance was measured at λ_max_ = 520 nm using a blank solution containing 4.9 mL distilled water, 0.5 mL of 0.1% HCl in ethanol and 10 mL of 2% HCl aqueous solution. The differences (ΔA) between the absorbance values of the samples were calculated. Thus, calculated (ΔA) values were multiplied by 875 in order to obtain the total anthocyanins content subsequently expressed in mg/L of DME [53].

### 3.8. Evaluation of Antioxidant Activity

The efficiency of the plant extract to neutralize DPPH (1,1-diphenyl-2-picrylhydrazyl radical) free radicals was determined using the spectrophotometric method [54]. From the plant extracts dissolved in methanol (1 mg/mL), dilutions were made to obtain concentrations of 500, 250, 125, 62.5, 31.25, 15.62, 7.81, 3.90, 1.99, and 0.97 mg/mL. Diluted solutions (1 mL each) were mixed with 1 mL of 80 mg/mL DPPH methanolic solution, and the absorbance was recorded at 517 nm (spectrophotometer Jenway UV/VIS 6105 with the resolution range 1 nm). The control samples contained methanol and DPPH reagents. The percentage inhibition was calculated using the equation: %inhibition = 100 × (A of control—A of sample)/A of control, while IC_50_ values were estimated based on the sigmoidal curve presenting the dependence of the percent of DPPH scavenging on sample concentration. Antioxidant activity was expressed as the half-maximal inhibitory concentration (IC_50_ values in mg/mL). In the presented results, antioxidant efficiency of the extract increased with decreasing IC_50_ values. The data were presented as mean values ± standard deviation (*n* = 3).

### 3.9. Evaluation of In Vitro Antimicrobial Activity

#### 3.9.1. Test Microorganisms

Antibacterial activity of the DME was tested against Gram-positive *Staphylococcus aureus* (ATCC 6538), *Bacillus cereus* (ATCC 11778), *Sarcina lutea* (ATCC 9431), and Gram-negative bacteria, the *Klebsiella oxytoca* (ATCC 8724), *Pseudomonas aeruginosa* (ATCC 9027), and *Salmonella enterica* (ATCC 13076). Testing of antifungal activity was performed against mold species *Aspergillus brasiliensis* (ATCC 16404). Bacterial strains were maintained on Nutrient Agar (NA) at optimal temperature of 37 °C, while the fungal strains were cultured on Sabouraud Dextrose Agar (SDA) at 30 °C at the Microbiology Laboratory of Department of Biology, Faculty of Science and Mathematics, University of Niš.

#### 3.9.2. Screening of Antimicrobial Activity (Microdilution Method)

Antimicrobial activity was evaluated using a broth microdilution method [55]. Bacterial suspensions were made from overnight cultures in Mueller–Hinton broth (mold suspension was made in SDB), and their turbidity was standardized to 0.5 McFarland using densitometer (DEN-1 McFarland Densitometer, Biosan). The final density of bacterial inoculums was 5 × 10^5^ CFU (colony forming units), while mold’s final inoculum size corresponded to 1 × 10^4^. Stock solutions of the DME were prepared in pure dimethyl sulfoxide (DMSO) and serially diluted (the diluting factor 2) with sterile saline in the concentration range 0.001–20.00 mg/mL. The highest final concentration of DMSO in each well was 10%. After making dilutions of the test substances, the inoculum was added to all wells and the plates were incubated at 37 °C during 24 h. Streptomycin and nystatin served as positive controls as antibacterial and antifungal agents, respectively, while one non-inoculated well, free of antimicrobial agent, was included to ensure the medium sterility. The bacterial growth was determined by adding 20 μL of 0.5% triphenyltetrazolium chloride (TTC) aqueous solution. MIC was defined as the lowest concentration of the test compound that inhibited visible growth (red colored pellet on the bottom of the wells after the addition of TTC). All experiments were done in triplicate.

### 3.10. Statistical Analysis

All experimental measurements were carried out in triplicate and are expressed as the average of three analyses ± standard deviation. Results were analyzed using IBM, SPSS, Statistics, ver. 19, Armonk, NY, USA: IBM Corp. Analysis of variance (ANOVA) and Tukey test with a level of confidence of 95% were performed for pairwise comparison of plant species in different analyses. Principal Component Analysis (PCA), Pearson Linear Correlation, and Agglomerate Hierarchical Clustering (AHC) using the Ward method were performed using XLSTAT for Windows (Addinsoft, New York, NY, USA) to visualize relationships among analyzed halophytic species.

## Figures and Tables

**Table 1:** Total phenolic content and flavonoid concentration in dry methanol (DME) and crude water (CWE) extracts of halophytes from different coastal saline habitats of Balkan Peninsula.

	Total Phenolic Content		Flavonoid Concentration	
Plant Species	DME mg of GA/g	CWE mg of GA/mL	DME mg of Ru/g of E	CWE mg of Ru/mL of I
*Pancratium maritimum*—Mn	81.65 ± 1.85 j	145.93 ± 0.78 l	24.17 ± 0.46 mn	20.53 ± 0.37 de
*Salsola kali*—Mn	84.42 ± 0.82 j	83.45 ± 0.72 p	66.53 ± 1.08 b	17.22 ± 0.19 g
*Salsola kali*—Al	62.99 ± 1.35 l	71.64 ± 0.36 q	69.47 ± 0.65 b	16.46 ± 0.09 hi
*Salsola kali*—Gr	32.51 ± 1.05 op	37.57 ± 0.74 w	34.47 ± 1.63 j	13.89 ± 0.03 k
*Salsola soda*—Mn	34.29 ± 0.98 op	89.05 ± 1.08 o	27.37 ± 0.78 kl	22.29 ± 0.06 c
*Salicornia europaea*—Mn	23.22 ± 1.53 q	45.59 ± 1.21 tu	22.61 ± 1.28 n	12.13 ± 0.21 m
*Sarcocornia fruticosa*—Mn	20.01 ± 1.14 q	51.24 ± 0.51 s	18.29 ± 0.49 o	16.95 ± 0.25 gh
*Halimione portulacoides*—Al	13.23 ± 1.62 r	43.65 ± 0.36 u	12.63 ± 1.12 p	16.50 ± 0.22 hi
*Halocnemum strobilaceum*—Al	31.60 ± 0.56 p	57.45 ± 0.81 r	26.82 ± 0.34 lm	17.35 ± 0.27 g
*Limonium angustifolium*—Mn	135.68 ± 1.66 g	233.89 ± 1.08 g	61.25 ± 0.79 c	20.37 ± 0.17 de
*Limonium vulgare*—Al	266.80 ± 0.39 c	511.10 ± 2.96 a	60.69 ± 0.52 c	26.35 ± 0.12 a
*Polygonum maritimum*—Mn	376.08 ± 2.49 a	363.08 ± 0.99 c	49.13 ± 0.39 fg	18.71 ± 0.09 f
*Tribulus terrestris*—Gr	31.74 ± 1.52 p	46.11 ± 0.93 stu	30.74 ± 1.79 k	12.42 ± 0.10 m
*Medicago marina*—Mn	20.11 ± 1.09 q	33.68 ± 0.70 w	27.94 ± 0.74 k	13.12 ± 0.12 l
*Euphorbia peplus*—Mn	186.73 ± 0.99 d	338.60 ± 1.03 d	58.34 ± 0.87 c	23.58 ± 0.27 b
*Cakile maritima*—Al	35.97 ± 1.22 op	90.85 ± 0.16 o	27.55 ± 0.89 kl	22.29 ± 0.21 c
*Alkanna tinctoria*—Al	164.42 ± 0.96 e	262.88 ± 1.07 f	50.68 ± 0.64 f	17.68 ± 0.20 g
*Centaurium maritimum*—Mn	45.24 ± 1.37 n	49.96 ± 1.07 st	42.19 ± 1.20 i	15.05 ± 0.07 j
*Vitex agnus-castus*—Gr	109.46 ± 0.97 h	216.96 ± 1.57 h	77.36 ± 0.61 a	26.10 ± 0.26 a
*Calystegia soldanella*—Mn	58.62 ± 1.50 m	159.74 ± 2.13 k	52.29 ± 0.33 f	23.80 ± 0.12 b
*Echinophora spinosa*—Mn	70.03 ± 1.32 k	210.27 ± 0.70 i	26.07 ± 0.95 kl	17.04 ± 0.38 gh
*Erygnium maritimum*—Mn	37.95 ± 1.17 o	118.46 ± 1.00 n	28.50 ± 1.51 k	13.95 ± 0.03 k
*Artemisia maritima*—Al	93.53 ± 1.56 i	277.78 ± 1.96 e	41.60 ± 0.24 i	21.16 ± 0.12 d
*Xanthium italicum*—Gr	48.59 ± 0.53 n	75.05 ± 1.07 q	34.35 ± 0.56 j	17.70 ± 0.35 g
* *Camelia sinensis*	356.06 ± 1.28 b	416.69 ± 4.32 b	58.22 ± 0.96 cd	18.82 ± 0.09 f
* Salvia officinalis	94.04 ± 0.85 i	134.69 ± 1.40 m	55.71 ± 0.55 cde	20.82 ± 0.03 d
* Lavandula officinalis	36.33 ± 0.18 op	209.65 ± 1.27 i	27.06 ± 1.09 lm	16.72 ± 0.10 hi
* Olea europaea	148.68 ± 0.50 f	178.67 ± 4.85 j	46.90 ± 0.97 gh	14.78 ± 0.07 j

* Commercially available plants used for comparation, Mn—Montenegro, Al—Albania, Gr—Greece. Means sharing the same letter in a column does not differ significantly by Tukey’s test (*p* ≥ 0.05).

**Table 2:** Content of total tannins and anthocyanins of halophytes from different coastal saline habitats of Balkan Peninsula.

Plant Species	Tannin Content g/L	Anthocyanin Content mg/L
*Pancratium maritimum*—Mn	0.72 ± 0.09 ghij	15.52 ± 0.43 c
*Salsola kali*—Mn	0.46 ± 0.05 ijk	12.22 ± 1.05 f
*Salsola kali*—Al	0.44 ± 0.05 ijk	15.76 ± 0.39 c
*Salsola kali*—Gr	0.49 ± 0.08 hijk	8.39 ± 0.38 ij
*Salsola soda*—Mn	0.57 ± 0.07 ghij	2.53 ± 0.48 m
*Salicornia europaea*—Mn	0.39 ± 0.02 jkl	7.82 ± 0.36 jk
*Sarcocornia fruticosa*—Mn	0.15 ± 0.04 kl	6.56 ± 0.43 k
*Halimione portulacoides*—Al	0.05 ± 0.01 l	4.54 ± 0.15 l
*Halocnemum strobilaceum*—Al	0.49 ± 0.02 hijk	7.38 ± 0.53 jk
*Limonium angustifolium*—Mn	0.88 ± 0.07 fg	10.65 ± 0.55 gh
*Limonium vulgare*—Al	0.90 ± 0.06 fg	8.30 ± 0.29 ij
*Polygonum maritimum*—Mn	0.75 ± 0.04 fghi	1.85 ± 0.13 m
*Tribulus terrestris*—Gr	2.36 ± 0.17 b	6.41 ± 0.34 k
*Medicago marina*—Mn	0.50 ± 0.07 hij	13.94 ± 0.35 de
*Euphorbia peplus*—Mn	1.77 ± 0.06 c	39.32 ± 1.14 a
*Cakile maritima*—Al	0.57 ± 0.07 ghij	7.38 ± 0.45 jk
*Alkanna tinctoria*—Al	0.89 ± 0.06 fg	30.90 ± 0.30 b
*Centaurium maritimum*—Mn	1.41 ± 0.12 def	15.72 ± 0.29 c
*Vitex agnus-castus*—Gr	2.47 ± 0.42 b	11.46 ± 0.49 fg
*Calystegia soldanella*—Mn	0.77 ± 0.08 fghi	31.22 ± 0.25 b
*Echinophora spinosa*—Mn	0.82 ± 0.08 fgh	7.39 ± 0.24 jk
*Erygnium maritimum*—Mn	1.15 ± 0.10 de	15.67 ± 0.40 c
*Artemisia maritima*—Al	0.58 ± 0.03 fghij	8.33 ± 0.32 ij
*Xanthium italicum*—Gr	3.50 ± 0.11 a	16.36 ± 0.26 c
* Camelia sinensis	2.59 ± 0.11 b	14.41 ± 0.39 cd
* Salvia officinalis	0.67 ± 0.05 ghij	9.43 ± 0.48 hi
* Lavandula officinalis	1.07 ± 0.04 f	10.54 ± 0.46 gh
* Olea europaea	2.39 ± 0.07 b	6.45 ± 0.39 k

* Commercially available plants used for comparation, Mn—Montenegro, Al—Albania, Gr—Greece. Means sharing the same letter in a column does not differ significantly by Tukey’s test (*p* ≥ 0.05).

**Table 3:** Antioxidant activity in dry methanol (DME) and crude water (CWE) extract of halophytes from different coastal saline habitats of Balkan Peninsula.

Plant Species	DWE IC_50_ (µg/mL)	CWE IC_50_ (µg/mL)
*Pancratium maritimum*—Mn	653.24 ± 0.81 r	393.02 ± 1.69 m
*Salsola kali*—Mn	224.25 ± 1.07 i	343.69 ± 2.33 l
*Salsola kali*—Al	178.96 ± 0.84 g	431.72 ± 1.46 n
*Salsola kali*—Gr	374.48 ± 2.15 m	1056.50 ± 2.45 v
*Salsola soda*—Mn	316.48 ± 1.52 k	296.80 ± 1.95 k
*Salicornia europaea*—Mn	947.88 ± 1.82 s	809.43 ± 1.92 s
*Sarcocornia fruticosa*—Mn	648.03 ± 1.63 q	699.23 ± 1.29 q
*Halimione portulacoides*—Al	1147.68 ± 2.17 u	1613.05 ± 2.09 x
*Halocnemum strobilaceum*—Al	458.02 ± 1.78 o	951.79 ± 1.35 u
*Limonium angustifolium*—Mn	58.66 ± 1.95 d	42.12 ± 0.78 b
*Limonium vulgare*—Al	15.52 ± 0.64 a	21.96 ± 0.89 a
*Polygonum maritimum*—Mn	15.02 ± 0.47 a	24.59 ± 0.91 a
*Tribulus terrestris*—Gr	462.97 ± 1.25 o	923.24 ± 2.04 t
*Medicago marina*—Mn	1025.44 ± 2.13 t	1386.67 ± 3.56 w
*Euphorbia peplus*—Mn	29.19 ± 1.61 b	26.98 ± 0.67 a
*Cakile maritima*—Al	392.50 ± 2.08 n	677.56 ± 1.14 p
*Alkanna tinctoria*—Al	52.86 ± 1.48 c	81.27 ± 0.94 cd
*Centaurium maritimum*—Mn	192.10 ± 1.88 h	804.16 ± 1.08 r
*Vitex agnus-castus*—Gr	58.40 ± 0.74 d	165.79 ± 0.68 h
*Calystegia soldanella*—Mn	349.67 ± 1.74 l	145.11 ± 1.21 g
*Echinophora spinosa*—Mn	249.62 ± 1.50 j	86.47 ± 1.41 e
*Erygnium maritimum*—Mn	474.04 ± 2.24 p	192.33 ± 1.32 j
*Artemisia maritima*—Al	91.52 ± 1.30 f	80.23 ± 1.01 c
*Xanthium italicum*—Gr	348.09 ± 1.27 l	521.98 ± 2.21 o
* Camelia sinensis	10.70 ± 0.86 a	24.05 ± 0.88 a
* Salvia officinalis	75.79 ± 0.72 e	173.27 ± 1.15 i
* Lavandula officinalis	254.26 ± 2.32 j	75.99 ± 1.36 c
* Olea europaea	61.77 ± 0.54 d	137.52 ± 1.44 f

* Commercially available plants used for comparation, Mn—Montenegro, Al—Albania, Gr—Greece. Means sharing the same letter in a column does not differ significantly by Tukey’s test (*p* ≥ 0.05).

**Table 4:** Antimicrobial activity (minimum inhibitory concentrations – MICs) of halophytes from different coastal saline habitats of Balkan Peninsula.

Plant/Microorganism Species	Gram-Positive Bacteria			Gram-Negative Bacteria			Mold
	*B. cereus*	*S. aureus*	*S. lutea*	*K. oxytoca*	*P. aeruginosa*	*S. enterica*	*A. brasiliensis*
*Pancratium maritimum*—Mn	2.50	2.50	10.0	10.0	2.50	5.0	10.0
*Salsola kali*—Mn	1.25	5.0	2.50	5.0	2.50	5.0	5.0
*Salsola kali*—Al	1.25	0.15	2.50	5.0	20.0	2.50	10.0
*Salsola kali*—Gr	5.0	5.0	5.0	10.0	10.0	5.0	5.0
*Salsola soda*—Mn	5.0	2.50	10.0	20.0	10.0	10.0	10.0
*Salicornia europaea*—Mn	5.0	10.0	20.0	10.0	5.0	10.0	20.0
*Sarcocornia fruticosa*—Mn	5.0	5.0	10.0	20.0	10.0	10.0	10.0
*Halimione portulacoides*—Al	5.0	10.0	10.0	10.0	10.0	10.0	10.0
*Halocnemum strobilaceum*—Al	10.0	10.0	10.0	10.0	10.0	10.0	10.0
*Limonium angustifolium*—Mn	2.50	0.15	0.15	1.25	1.25	0.31	0.31
*Limonium vulgare*—Al	0.31	0.15	0.62	0.15	0.15	0.31	0.31
*Polygonum maritimum*—Mn	2.50	2.50	2.50	0.15	5.0	0.31	5.0
*Tribulus terrestris*—Gr	0.62	10.0	10.0	10.0	10.0	10.0	1.25
*Medicago marina*—Mn	2.50	10.0	20.0	10.0	1.25	10.0	10.0
*Euphorbia peplus*—Mn	0.31	0.15	2.50	2.50	5.0	0.15	5.0
*Cakile maritima*—Al	5.0	5.0	10.0	20.0	10.0	10.0	10.0
*Alkanna tinctoria*—Al	1.25	0.62	0.31	5.0	2.50	2.50	5.0
*Centaurium maritimum*—Mn	10.0	10.0	5.0	20.0	10.0	2.50	20.0
*Vitex agnus-castus*—Gr	0.15	5.0	0.62	2.50	0.15	0.62	5.0
*Calystegia soldanella*—Mn	2.50	5.0	10.0	2.50	10.0	5.0	10.0
*Echinophora spinosa*—Mn	2.50	2.50	10.0	10.0	2.50	10.0	10.0
*Erygnium maritimum*—Mn	1.25	2.50	10.0	5.0	10.0	10.0	10.0
*Artemisia maritima*—Al	1.25	1.25	1.25	1.25	1.25	2.50	5.0
*Xanthium italicum*—Gr	5.0	1.25	0.31	1.25	10.0	0.31	2.50
* Camelia sinensis	2.50	2.50	5.0	5.0	5.0	5.0	10.0
* Salvia officinalis	0.62	0.62	5.0	0.31	0.31	0.15	1.25
* Lavandula officinalis	10.0	10.0	10.0	10.0	10.0	1.25	10.0
* Olea europaea	2.50	0.31	10.0	20.0	0.62	0.62	5.0
^#^ Streptomycin	0.09	0.09	0.09	0.09	0.09	0.09	/
^#^ Chloramphenicol	0.19	0.39	0.78	0.39	0.19	0.04	/
^#^ Nystatin	/	/	/	/	/	/	1.92

* Commercially available plants used for comparation; # antibiotics used as a positive control; Mn—Montenegro, Al—Albania, Gr—Greece.

**Table 5:** The main geographical data of the sampled coastal halophytes localities.

	Plant Family/Species	Site	GPS Data	Voucher Specimen
	Amaryllidaceae			
1	*Pancratium maritimum* L.—Mn	Velika plaža near Ulcinj	41°54′29.1″ N 19°14′52.1″ E	050613-5
	Amaranthaceae			
2	*Salsola kali* L.—Mn	Velika plaža near Ulcinj	41°54′18.6″ N 19°15′35.5″ E	050613-4
3	*Salsola kali* L.—Al	Greth area	41°03′16.7″ N 19°27′06.8″ E	060613-1
4	*Salsola kali* L.—Gr	Kalithea	40°06′107″ N 23°46′32.9″ E	150814-2
5	*Salsola soda* L.—Mn	Ulcinj Saline	41°55′08.6″ N 19°15′19.8″ E	050613-6
6	*Salicornia europaea* L.—Mn	Ulcinj Saline	41°55′07.6″ N 19°15′16.1″ E	050613-7
7	*Sarcocornia fruticosa* (L.) A.J.Scott—Mn	Tivat Saline	42°23′29.5″ N 18°42′46.0″ E	030613-9
8	*Halimione portulacoides* (L.) Aellen—Al	Greth area, laguna	41°01′02.1″ N 19°26′36.8″ E	060613-2
9	*Halocnemum strobilaceum* (Pall.) M.Bieb.—Al	New delta near Shumbini	41°02′24.6″ N 19°27′00.3″ E	070613-1
	Plumbaginaceae			
10	*Limonium angustifolium* (Tausch) Turrill—Mn	Tivat Saline	42°23′31.6″ N 18°42′42.2″ E	030613-10
11	*Limonium vulgare* Mill.—Al	Greth area, Shkumbini delta	41°02′24.6″ N 19°27′00.3″ E	060613-3
	Polygonaceae			
12	*Polygonum maritimum* L.—Mn	Tivat Saline	42°23′29.8″ N 18°42′51.4″ E	030613-8
	Zygophyllaceae			
13	*Tribulus terrestris* L.—Gr	Kalithea	40°06′36.06″ N 23°46′10.8″ E	150814-3
	Fabaceae			
14	*Medicago marina* L.—Mn	Velika plaža near Ulcinj	41°54′29.1″ N 19°14′52.1″ E	050613-3
	Euphorbiaceae			
15	*Euphorbia peplus* L.—Mn	Velika plaža near Ulcinj	41°54′29.1″ N 19°14′52.1″ E	050613-2
	Brassicaceae			
16	*Cakile maritima* Scop.—Al	Greth area, Shkumbini delta, sandy beach	41°03′00.8″ N 19°26′58.2″ E	060613-4
	Boraginaceae			
17	*Alkanna tinctoria* (L.) Tausch—Al	Greth area, Shkumbini delta, near last sandy beach belt to shrub-forest	41°03′05.0″ N 19°27′05.8″ E	060613-6
	Gentianaceae			
18	*Centaurium maritimum* (L.) Fritsch—Mn	Velika plaža near Ulcinj	41°54′18.6″ N 19°15′35.5″ E	050613-1
	Lamiaceae			
19	*Vitex agnus-castus* L.—Gr	Kalithea	40°06′13.9″ N 23°46′21.2″ E	150814-4
	Convolvulaceae			
20	*Calystegia soldanella* (L.) R.Br.—Mn	Velika plaža near Ulcinj	41°54′18.6″ N 19°15′35.5″ E	040613-3
	Apiaceae			
21	*Echinophora spinosa* L.—Mn	Velika plaža near Ulcinj	41°54′18.6″ N 19°15′35.5″ E	040613-1
22	*Erygnium maritimum* L.—Mn	Velika plaža near Ulcinj	41°54′18.6″ N 19°15′35.5″ E	040613-2
	Asteraceae			
23	*Artemisia maritima* L.—Al	Greth area, Shkumbini delta	41°03′02.7″ N 19°27′05.2″ E	060613-5
24	*Xanthium italicum* Moretti—Gr	Kalithea	40°06′13.9″ N 23°46′21.2″ E	150814-5

## Data Availability

Not applicable.
